# Edge4TSC: Binary Distribution Tree-Enabled Time Series Classification in Edge Environment

**DOI:** 10.3390/s20071908

**Published:** 2020-03-30

**Authors:** Chao Ma, Xiaochuan Shi, Wei Li, Weiping Zhu

**Affiliations:** 1 School of Cyber Science and Engineering, Wuhan University, Wuhan 430079, China; chaoma@whu.edu.cn (C.M.); shixiaochuan@whu.edu.cn (X.S.); auto_weili@whu.edu.cn (W.L.); 2 School of Computer Science, Wuhan University, Wuhan 430079, China

**Keywords:** time series classification, edge environment, binary distribution tree, deep learning

## Abstract

In the past decade, time series data have been generated from various fields at a rapid speed, which offers a huge opportunity for mining valuable knowledge. As a typical task of time series mining, Time Series Classification (TSC) has attracted lots of attention from both researchers and domain experts due to its broad applications ranging from human activity recognition to smart city governance. Specifically, there is an increasing requirement for performing classification tasks on diverse types of time series data in a timely manner without costly hand-crafting feature engineering. Therefore, in this paper, we propose a framework named Edge4TSC that allows time series to be processed in the edge environment, so that the classification results can be instantly returned to the end-users. Meanwhile, to get rid of the costly hand-crafting feature engineering process, deep learning techniques are applied for automatic feature extraction, which shows competitive or even superior performance compared to state-of-the-art TSC solutions. However, because time series presents complex patterns, even deep learning models are not capable of achieving satisfactory classification accuracy, which motivated us to explore new time series representation methods to help classifiers further improve the classification accuracy. In the proposed framework Edge4TSC, by building the binary distribution tree, a new time series representation method was designed for addressing the classification accuracy concern in TSC tasks. By conducting comprehensive experiments on six challenging time series datasets in the edge environment, the potential of the proposed framework for its generalization ability and classification accuracy improvement is firmly validated with a number of helpful insights.

## 1. Introduction

In the past decade, the time series data are generated from various domains at a rapid speed [1], which offers a huge opportunity for mining valuable knowledge. As a typical task of time series mining, Time Series Classification (TSC) has attracted lots of attention from both researchers and domain experts due to its broad applications, such as human activity recognition [2], clinical data analysis [3], wind power forecasting [4], psychological research [5], complex event detection [6,7] and conjunctivities classification [8].

Recently, there has been an increasing requirement for performing classification tasks on diverse types of time series data in a timely manner. As we all know, computation and storage resources of most end devices are very limited, which makes locally processing collected data impractical. So, in most cases, raw time series data will be sent to the remote server for further processing. However, the unstable network status is a big challenge for such processing mode, especially for large volume of sensor data. Fortunately, edge computing [9], as an emerging technique, has become the reasonable choice to cope with such challenge. It imposes the intensive computation on edge devices that are located much closer to end devices than the remote server. Therefore, in this paper, we propose a framework that allows time series to be processed on the edge device so that the classification results can be instantly returned to end-users.

To solve TSC problems, the 1-Nearest-Neighbor classifier (denoted as 1NN) based on Euclidean distance [10] is usually selected as a baseline. And 1NN Dynamic Time Warping (denoted as DTW) is another TSC approach proposed by Reference [11], which aims to search for an optimal match between two given time series. Besides, many feature-based [12,13,14,15] and bag-of-pattern based [16,17,18,19] TSC solutions are proposed with various strategies for extracting discriminative features. However, domain knowledge is usually required for extracting discriminative features, which can be time-consuming and varies among different experts. Therefore, deep learning models have recently been applied to TSC problems [20].

Although there are many TSC solutions to cope with datasets presenting complex patterns, existing solutions are not able to achieve satisfactory accuracy. This indicates that the limited classification accuracy is probably caused by the inappropriate time series representation rather than the classifier design. Hence, we propose a new time series representation method based on the binary distribution tree, with the hope that it can help existing TSC classifiers further improve their classification accuracy.

The contributions of this paper mainly focus on the following three aspects:

- A new framework Edge4TSC that allows time series to be processed on the edge device.
- A new time series representation method based on binary distribution tree which transforms the original time series into the hierarchical distribution space.
- Comprehensive experiments on 6 challenging time series datasets with insightful analysis about the impact of key factors on the classification accuracy of 4 classifiers.

The rest of this paper is organized as follows. The TSC problem is formally defined in Section 2. In Section 3, the methodology is illustrated in detail, including the overall architecture of Edge4TSC, the binary distribution tree-based representation method, and deep learning-based classifiers. In Section 4, comprehensive experiments are conducted on 6 challenging datasets with a thorough analysis of the impact of key factors. Related works on existing TSC solutions are discussed in Section 5, followed by the conclusion made in Section 6.

## 2. Problem Formulation

The TSC problem can be formulated as follows.

**Given:**

- a set of class labels $C=\left\{c_{k}\right\}\left(1\leq k\leq K\right)$;
- a train set of time series $TS_{train}=\left\{ts_{train}^{p}\right\}\left(1\leq p\leq P\right)$ in which each time series $ts_{train}^{p}$ is attached with one class label $c_{k}\in C$; and
- a test set of time series $TS_{test}=\left\{ts_{test}^{q}\right\}\left(1\leq q\leq Q\right)$ in which each time series $ts_{test}^{q}$ is attached with one class label that is unknown during classification but available for evaluation;

**assume:**

- $TS_{train}\cap TS_{test}=\emptyset$;
- each time series $ts=\left\{tpv_{i}\right\}\left(1\leq i\leq N,ts\in TS_{train}\cup TS_{test}\right)$ consists of a set of consecutive numerical values (i.e., the time point value $tpv_{i}$ must be a real number); and
- the class label of each time series $ts_{test}^{q}\in TS_{test}$ must be included in the class label set *C*.

**Objective:**

- Maximize the classification accuracy $Acc_{F}^{R}$ defined as follows:

$$Acc_{F}^{R}=\frac{\sum_{q=1}^{Q}hit_{q}}{Q},$$

where $hit_{q}$ is 1 if the class label of the time series $ts_{test}^{q}\in TS_{test}$ is correctly predicted by the classifier *F* using representation *R*; otherwise, $hit_{q}$ is 0.

## 3. Methodology

### 3.1. Overall Architecture

**Figure 1:**
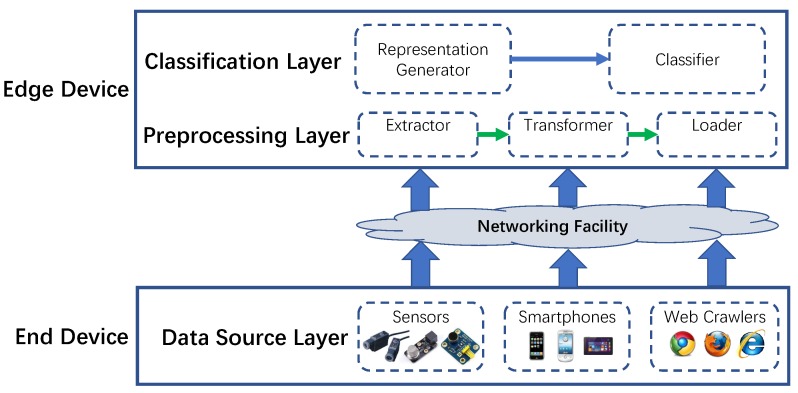
Overall architecture of Edge4TSC. TSC = Time Series Classification.

To meet the increasing requirement of performing classification tasks on diverse types of time series data in a timely manner without costly hand-crafting feature engineering, we propose a framework named Edge4TSC that allows the time series data to be classified on the edge device rather than the remote server. The framework consists of modules deployed on the end device and edge device, respectively. On the end device, the Data Source Layer implemented on data sources, such as sensors, smartphones, or web crawlers, is mainly responsible for collecting series-like raw data. Then, the raw time series will be sent to processing modules deployed on the edge device via the networking facility by using various communication techniques (e.g., WiFi, Bluetooth, Zigbee, 5G, etc.). Afterwards, the raw time series will first undergo the extract-transform-load (ETL) process, which is supported by modules of the Preprocessing Layer. Finally, the cleaned time series are fed into the Classification Layer. In the Classification Layer, the Representation Generator aims to produce the new representation as the input to the Classifier. And the Classifier is responsible for generating the final classification results. The overall architecture of the proposed framework is shown in Figure 1. In this paper, since we utilize the standard time series datasets for validation and thus do not need to consider data collection, the focus will be on the Classification Layer. Hence, in the following sub-sections, Section 3.2 and Section 3.3, a new time series representation method and the classifier design are illustrated in detail.

### 3.2. Time Series Representation Based on Binary Distribution Tree

In this paper, by utilizing Binary Distribution Tree (BDT), an approach to generating new time series representation is proposed. The entire procedure for obtaining the BDT-based representation consists of three main stages: Binary Subsequence Tree (BST) construction, Binary Distribution Tree (BDT) transformation, and representation generation. At the first stage, the goal is to build the Binary Subsequence Tree (BST) of which nodes at the same level contain subsequences of the original time series with no intersection. Given the time series ts and the split ratio sr, which is a decimal between 0 and 1 that helps decide where to cut the time series of the current node into two non-intersected subsequences, the way for constructing BST could be generally depicted as following steps: 1) set the original time series ts as the root node of the BST, and return BST if ts has only one element; 2) calculate the split position sp=integer(len(ts)*sr) (len(ts) is the length of ts) for the current node, and return BST if sp is 1; 3) add the subsequences $ts_{1,sp}$ and $ts_{sp+1,len(ts)}$ as the left and right child nodes of the current node; 4) step 2 and 3 would be conducted in a recursive manner until the entire BST is constructed. A more formal description of BST construction on time series ts with the split ratio sr is provided, as shown in Algorithm 1.
**Algorithm 1** Build_BST**Input:**ts,sr in **Output:**
BST(ts) out *Initialisation*:
1:$BST{\left(ts\right)}_{root}^{root}=ts$2:**if** (len(ts)==1) **then**3:  **return** BST(ts)
4:**end if**
 *Iterative Process*:
5:sp=integer(len(ts)*sr)6:**if** (sp==1) **then**7:  **return** BST(ts)
8:**end if**9:left_series=ts[1,sp]10:right_series=ts[sp+1,len(ts)]11:$BST{\left(ts\right)}_{root}^{left}=Build\_BST\left(left\_series,sr\right)$12:$BST{\left(ts\right)}_{root}^{right}=Build\_BST\left(right\_series,sr\right)$
 *Output Binary Subsequence Tree for ts*:
13:**return**BST(ts)


**Figure 2:**
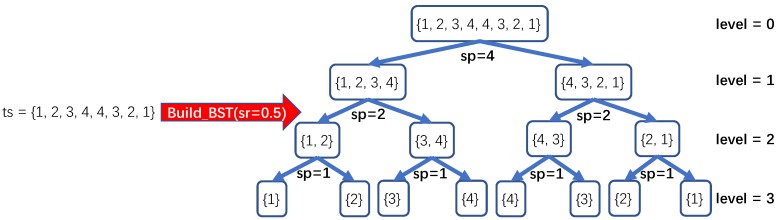
An example for Binary Subsequence Tree (BST) construction procedure.

To illustrate the procedure for constructing the binary subsequence tree (i.e., BST) in detail, we would like to utilize a concrete example, as shown in Figure 2. Suppose it is given a time series ts={1,2,3,4,4,3,2,1} and the split ratio sr=0.5, which means that each time we would like to split the current time series into two subsequences at the middle position. In this example, we would like to use the depth-first method for iteratively creating nodes of BST. Firstly, ts is set as the root of BST, which is the only node at level 0 of BST. And then the split position sp is calculated, which is the integer part of the result by multiplying the length of ts 8 and the split ratio sr 0.5. And now, in this operation, the split position sp is 4. The parent time series ts is divided into a subsequence containing the first four elements {1,2,3,4} of ts and a subsequence containing the other four elements {4,3,2,1} of ts. Afterwards, the former subsequence is set as the left child node of the root, while the latter becomes the right child node of the root. And due to the adoption of the depth-first strategy, we set the left node of the root as the current node and find it contains more than one element, which means that the split procedure will continue. Thus, we calculate the split position sp for the current node, which is 2 according to the length of {1,2,3,4} and the split ratio sr=0.5. Similarly, the first two elements {1,2} and the rest two elements {3,4} become the left and right child nodes of the current node (i.e., the node {1,2,3,4}). After that, the node {1,2} is set as the current node with the split position sp computed as 1. Finally, nodes {1} and {2} are recorded as the left and right child nodes of the current node {1,2}, which indicates the termination for splitting on both newly created nodes due to the fact that both nodes have only one element. And then we go back to other leaf nodes which have more than one element and apply the aforementioned procedure to them for constructing the complete BST, as shown in Figure 2.

Once the BST construction process is done, the second stage could be launched for generating the Binary Distribution Tree for ts based on BST. As we know, to avoid the so-called data leakage problem, it is forbidden to use any information or data sample from the test set during the training process. This is also applicable to the process for generating new representations for time series. Thus, no information nor data sample from the test set will be used for generating new representations. Rather than using the original data points of the time series and its subsequences, transforming them into the hierarchical distribution space could be helpful for not only reserving the global distribution but also presenting the local distribution for the original time series. Given BST(ts), $TS_{train}$, and bins (an integer greater than 1 to help determine the bin edges which are critical for gaining the distribution of the given subsequence), the way for constructing the binary distribution tree BDT(ts) for ts could be generally depicted as follows: 1) compute the bin_width according to the maximum, minimum of $TS_{train}$ and bins; 2) determine the bin_edges according to the minimum of $TS_{train}$ and the bin_width (each bin has a range of the same width); and 3) traverse BST(ts) and transform the subsequence in each node of BST(ts) into the distribution format by computing its histograms over bin_edges. A more formal description of the binary distribution tree construction on the time series ts is provided, as shown in Algorithm 2.
**Algorithm 2** Build_BDT**Input:**$BST\left(ts\right),TS_{train},bins$ in
**Output:**
BDT(ts) out *Initialisation*:
1:$minimum=argmin(TS_{train})$2:$maximum=argmax(TS_{train})$3:bin_width=(maximum-minimum)/bins
 *Determining Bin Edges*:
4:bin_edges[1]=minimum5:**for**i=1 to bins **do**6: bin_edges[i+1]=bin_edges[i]+bin_width
7:**end for**
 *Transforming BST(ts) into Distribution Space*:
8:**for**eachnode in BST(ts) **do**9: $BDT{\left(ts\right)}_{node}=histogram\left(BST{\left(ts\right)}_{node},bin\_edges\right)$
10:**end for**
 *Output Binary Distribution Tree for ts*:
11:**return**BDT(ts)


**Figure 3:**
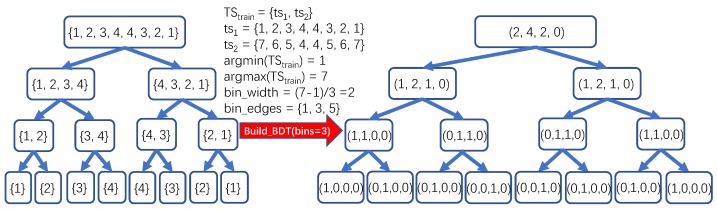
An example for Binary Distribution Tree (BDT) transformation procedure.

To illustrate the procedure for transforming the binary subsequence tree (i.e., BST) to binary distribution tree (i.e., BDT) in detail, we would like to utilize a concrete example, as shown in Figure 3. Suppose it is given the train set $TS_{train}=\left\{ts_{1},ts_{2}\right\}$ containing two time series $ts_{1}=\left\{1,2,3,4,4,3,2,1\right\}$ and $ts_{2}=\left\{7,6,5,4,4,5,6,7\right\}$, and the value of bins which is set to 3. The whole procedure of transforming BST to BDT consists of two phases. As shown in Figure 3, above the red arrow, the first step of the first phase is to determine the minimum and maximum of the train set $TS_{train}$. Obviously, by executing the functions $argmin(TS_{train})$ and $argmax(TS_{train})$, 1 and 7 are obtained as the minimum and maximum of $TS_{train}$, respectively. At the second step of the first phase, the gap between the minimum 1 and maximum 7 is computed as 6. To divide by bins (set to 3 in this example), the bin_width is computed as 2, which means that each bin is of the width 2. By the end of the first phase, bin_edges={1,3,5} records the edge for each bin with the width 2. At the second phase, each BST node is transformed to a histogram based format according to the number of elements falling into the specific bin defined by bin_edges. For instance, the BST root is {1,2,3,4,4,3,2,1}, having 2 elements ({1,1}) in range (-∞,1], 4 elements ({2,3,2,3}) in range (1,3], 2 elements ({4,4}) in range (3,5], and 0 element in range (5,+∞). Hence, the histogram based representation for the BST root is (2,4,2,0). The histogram based representations for the rest nodes of BST are shown in Figure 3.

**Figure 4:**
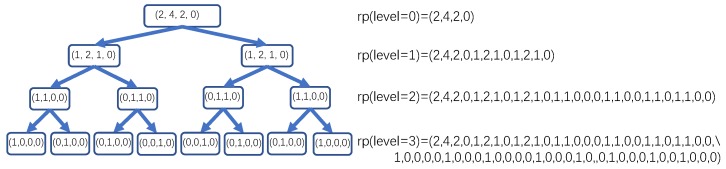
An example for BDT-based representation generation.

When BST construction and BDT transformation are finished, the final stage to obtain the BDT-based representation of ts is to concatenate each node from level 0 to the target level of BDT(ts) as a vector in a width-first order. For BDT shown in Figure 3, the BDT-based representations of all levels are shown in Figure 4. rp(level=x) represents the representation by concatenating the nodes from level 0 to level x of the binary distribution tree (i.e., BDT) in a width-first order. Therefore, for the BDT-based representation method, three parameters (i.e., sr, bins, and level) described in Table 1 work jointly to uniquely identify a specific representation. By executing BST construction, BDT transformation, and BDT-based representation generation on all original time series from both train and test sets, the original time series are mapped from the raw representation space to the BDT-based representation space and are now ready to be fed into the classifier for training and classification.

### 3.3. Classifier Selection and Design

When the representation of time series is ready, the next step is to select a proper classifier. In this paper, the 1NN classifier based on Euclidean distance is selected because it is of simple implementation and needs no parameter tuning. Therefore, the effect, if any, on the classification performance of 1NN classifier using the proposed BDT-based representation can be easily identified. For the 1NN classifier, it calculates the Euclidean distance between the test time series and all the train time series. Then, it predicts the label of the test time series as the label of the train time series that has shorter distance to that test time series than all the other train time series.

**Figure 5:**
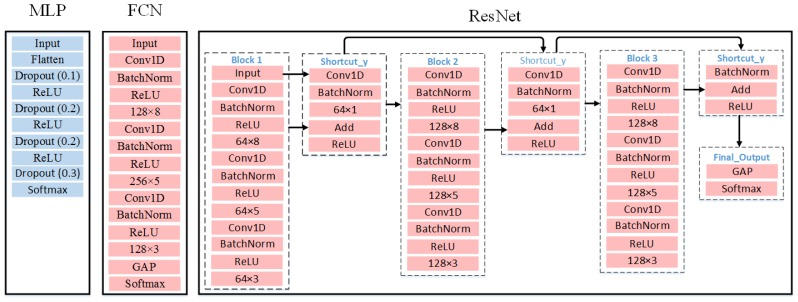
Network structure and hyper-parameter setting of deep learning classifiers. MLP = Multi-Layer Perceptron; FCN = Fully Convolutional Network; ResNet = Residual Network; ReLU = Rectified Linear Unit; GAP = Global Average Pooling.

Meanwhile, as deep learning models show promising potential for solving TSC problems [20], we are very interested in recruiting three deep learning models, Multi-Layer Perceptron (MLP), Fully Convolutional Network (FCN), and Residual Network (ResNet), as the candidate classifiers for validating the effectiveness of the BDT-based representation method. For all the three deep learning classifiers, the Input layer is to receive the time series representation as the input. And the Softmax layer is responsible for mapping the classification results to different class labels. Furthermore, the Rectified Linear Unit (ReLU layer) is adopted as the activation function for all deep learning classifiers. To avoid overfitting in MLP, the Dropout technique (Dropout layer) is employed where the decimal indicates the probability of randomly dropping the weights. For FCN and ResNet, the Conv1D layer is utilized for conducting one dimensional convolution operations. In addition, the Batch Normalization technique (BatchNorm layer) is applied to FCN and ResNet, which leads to faster learning rates. The purpose of employing the Global Average Pooling (GAP layer) in FCN and ResNet is to dramatically reduce the amount of parameters, thus speeding up the training process. The network structure and the hyper-parameter setting of MLP, FCN, and ResNet are shown in Figure 5.

## 4. Evaluation

### 4.1. Experimental Settings

To emulate the edge environment, a laptop is employed as the data source where consecutive data points of each time series are sent to a mobile work station, which plays the role of the edge device. The time interval for sending consecutive data points is set to 1ms, so that the time series with a length shorter than 1000 could be sent to the edge device within one second. For deep learning classifiers, all time series in the train set will be transmitted to the edge device via WiFi network for classifier training, and then time series in the test set will be fed into the well-trained classifiers for label prediction. For the 1NN classifier, there will be no model training process. The class label of time series in the test set would be predicted by calculating the Euclidean distance between the test time series and all train set time series.

For validating TSC solutions, the most widely used time series datasets collected from different application fields are archived by Reference [21] and are called UCR Archive. Although there are more than 80 datasets in the UCR Archive, we selected 6 challenging datasets on which neither 1NN Euclidean classifier nor deep learning models are able to achieve satisfactory classification accuracy. More details about these 6 datasets are provided in Table 2.

As explained in Table 1, there are three parameters (i.e., sr, bins, and level) to determine a BDT-based time series representation. In our experiments, sr is initialized as 0.1 and increased by 0.1 until it reaches 0.9 (for deep learning classifiers due to their time-consuming training process), or initialized as 0.05 and increased by 0.05 until it reaches 0.95 (for 1NN Euclidean classifier due to its low computation cost). bins is initialized as 3 and increased by 1 until it reaches 20 (for deep learning classifiers due to the same reason as sr), or until it reaches 30 (for 1NN Euclidean classifier due to the same reason as sr). level is set to 0 initially and increased by 1 until it reaches 9. Since the 1NN classifier is a deterministic model, we train the 1NN classifier for only one time on each representation and record the classification accuracy. Different from the 1NN classifier, the outputs of deep learning classifiers are not deterministic. Thus, we run 10 iterations for each deep learning classifier on each representation and take the average accuracy of 10 runs as the final performance. The training process of deep learning classifiers will last for 100 epochs with the batch size set to 16. One more thing to note is that the accuracy of the deep learning classifiers on the test set is taken at the epoch when the loss on the train set reaches the lowest.

### 4.2. Overall Results and Analysis

The experimental results of 4 selected classifiers over 6 challenging datasets with and without the BDT-based representation are shown in Table 3. "RAW" or "BDT" means that the original representation or the BDT-based representation of the time series is fed to the specific classifier. The accuracy is in bold if it outperforms the couterpart. Obviously, among all the six datasets, the 1NN Euclidean classifier using BDT-based representation outperforms its counterparts with the raw representation, and the classification accuracy is significantly enhanced from 0.377 to 0.466 on average.

For MLP, BDT-based representation helps improve the performance on all the 6 datasets (5 wins and 1 tie), and the average accuracy is improved from 0.409 to 0.483 on average. When we check the results of ResNet and FCN, it is observed that, for 4 out of 6 datasets, the TSC accuracy is significantly improved, while the average accuracy is boosted to 0.481 and 0.46, respectively. According to the overall experimental results, it could be concluded that the accuracy enhancement is achieved by the adoption of BDT-based representation, since all classifiers have exactly the same configuration and training strategy.

**Figure 6:**
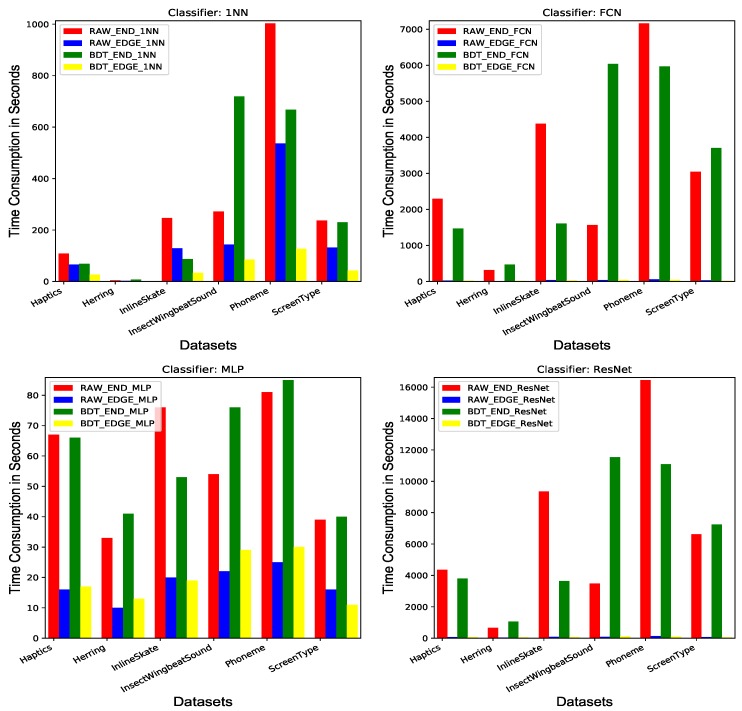
Time consumption of end device and edge device. 1NN = 1-Nearest-Neighbor.

To demonstrate the benefits of utilizing the edge device to train the classifiers, we show the time consumption of the edge device and the end device for training different classifiers over all the six datasets in Figure 6. For the 1NN classifier, it can only utilize the CPU of either the end device or the edge device. For the other three deep learning classifiers, MLP, FCN, and ResNet, they can be trained efficiently on the edge device equipped with a GPU. The three parts of the legend in Figure 6 indicate the representation method (RAW/BDT), the device (END/EDGE), and the classifier (1NN/MLP/FCN/ResNet). For instance, the legend RAW_END_1NN represents the time consumption of running the 1NN classifier using the raw representation of the specific dataset on the end device. As can be observed in Figure 6, for both representation methods RAW and BDT, it saves a lot of time by offloading the classifier training process from the end device to the edge device. And such benefit is more dramatic for FCN and ResNet than 1NN and MLP. The possible reason might be that 1NN cannot utilize the GPU of the edge device for further efficiency enhancement. Thus, the efficiency improvement of 1NN classifier is mainly due to the more powerful CPU of the edge device than the end device. And for MLP, it has much fewer tunable parameters than FCN and ResNet, thus benefiting less from the great parallelism of the edge device GPU. Obviously, from FCN and ResNet charts in Figure 6 where the time consumption of the edge device can hardly be observed, FCN and ResNet obtain the tremendous speed-up by fully utilizing the GPU of the edge device for updating massive parameters in high parallelism. According to the above-mentioned reasons, it is valuable to employ Edge4TSC framework for building time series classification systems, especially when heavy classifiers, such as FCN or ResNet, get involved. Although the average time consumption of the edge device on a single representation is reasonably affordable, the entire process for searching the optimal representation in the given space could be as long as several days or even several weeks.

Since each unique BDT-based representation is generated by using three parameters, sr, bins, and level, it will be helpful for exploring their impact on TSC accuracy of each selected classifier, which is investigated separately in Section 4.3, Section 4.4 and Section 4.5.

### 4.3. Impact of Split Ratio on TSC Accuracy

**Figure 7:**
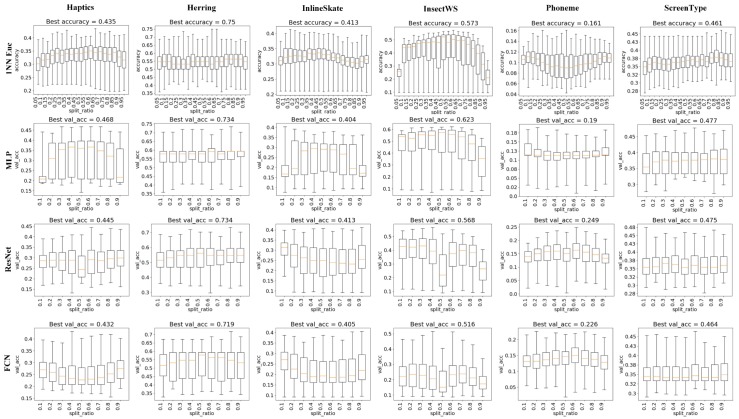
Impact of split ratio on TSC accuracy.

**Figure 8:**
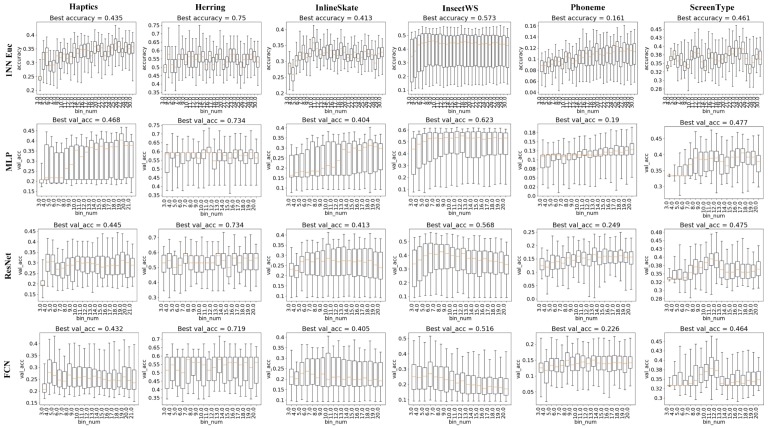
Impact of bin number on TSC accuracy.

**Figure 9:**
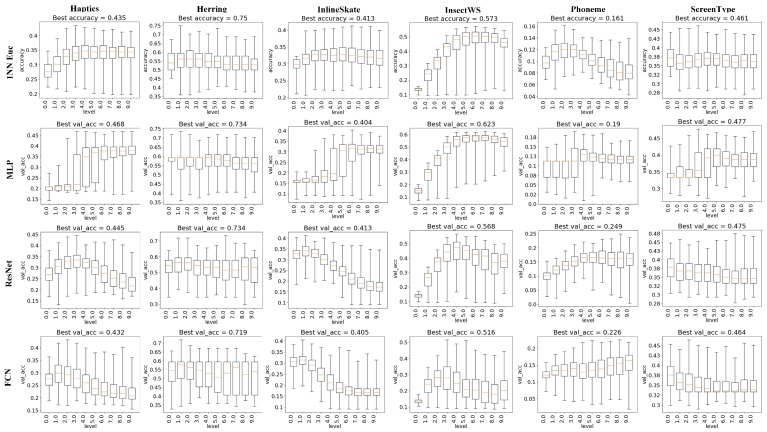
Impact of BDT level on TSC accuracy.

To make understanding the experimental results easier and more accurate, we would like to take an example to illustrate the detailed process of generating results, as shown in Figure 7, Figure 8 and Figure 9, in advance. As mentioned in Section 4.1, for deep learning models, the split ratio sr ranges from 0.1 to 0.9 with a stride 0.1 and the bin number bins ranges from 3 to 20 with a stride 1, which means that there will be 9×18 binary distribution trees in total (9 different split ratios and 18 different bin number values). And for each binary distribution tree, we would generate 10 representations from level 0 representation to level 9 representation. Therefore, for each dataset, the amount of different representations will be 9×18×10. Furthermore, since deep learning classifiers may generate varied results due to their random parameter initialization strategy, we conduct 10 runs for the same representation and take the average accuracy as the final performance for that representation. Let us take the chart at row 2 and column 1 in Figure 7 as an instance, which shows the impact of split ratio on dataset Haptics adopting classifier MLP. In that chart, MLP is trained and tested on 9×18×10 BDT-based representations. All the 9×18×10 accuracy results are grouped into 9 sets by the split ratio, while each set contains 18×10 accuracy results with the same split ratio. Then, 9 boxes are plotted for 9 sets with different split ratios. Each box extends from the lower to upper quartile values of the classification accuracy on 18*10 BDT-based representations with the split ratio corresponding to the x-axis tick, with a colored line at the median. The whiskers extend from the box to show the range of the classification accuracy. The top short horizontal line represents the highest classification accuracy among representations in the same group, while the bottom short horizontal line represents the lowest. Therefore, by analyzing the boxes among different x-axis values (in this case, that is the split ratio ranging from 0.1 to 0.9 with a stride 0.1), it is easier to figure out the quantitative impact of the specific factor (i.e., the split ratio in Figure 7) on the y-axis variable (i.e., the classification accuracy).

The experimental results about the impact of split ratio sr on TSC accuracy are shown in Figure 7 as box plots, with minimum, maximum, and average classification accuracy from comprehensive experiments. The six columns represent 6 datasets and the four rows represent 4 classifiers. For 1NN Euclidean classifier, the best accuracy is usually observed when sr is 0.4, 0.5 or 0.6. In particular, on the dataset "InsectWingbeatSound", both best accuracy and average accuracy increases until sr reaches 0.6 and then decreases afterwards. A similar phenomenon is also observed in MLP cases. However, in ResNet and FCN cases, it becomes quite different, as both best accuracy and average accuracy are significantly lower than most cases when sr is 0.5. The impact of sr on ResNet and FCN looks similar to each other, while 1NN Euclidean classifier and MLP share some common trends.

### 4.4. Impact of Bin Number on TSC Accuracy

For the parameter bins, intuitively, a rather small bins tends to make the BDT-based representation undiscriminating among times series, especially those with many categories. When a rather large bins is set, it would make the BDT-based representation too sensitive to filter out the negative effect of noisy data points in the original time series. To study the impact of bins on classification accuracy, experiments were conducted, and detailed results are shown in Figure 8. And it is clearly seen that almost all classifiers on all tested datasets benefit from a larger value of bins to different extents. And when bins becomes larger than a certain threshold, either the best accuracy or the average accuracy tend to fluctuate (e.g., 1NN Euclidean on ScreenType when sr is greater than 12) or even decrease (e.g., 1NN Euclidean on InlineSkate when sr is greater than 10).

### 4.5. Impact of BDT Level on TSC Accuracy

To obtain the BDT-based representation of time series, the nodes from the BDT root to level of BDT are concatenated. Therefore, the deeper level is, the more detailed information of the original time series would be contained by the BDT-based representation. The experimental results that helped us quantify the impact of level over classification accuracy are shown in Figure 9. It is found that 1NN Euclidean and MLP classifiers could benefit from the adoption of BDT-based representations when level increases except on dataset "Herring". And it seems that ResNet and FCN models prefer a relatively small level, such as 2, 3, or 4.

## 5. Related Work

For solving TSC problems, the most frequently adopted baseline is probably the 1NN classifier based on Euclidean distance (denoted as 1NN-EUC) due to its parametric-free and time-efficient features [10]. Then, inspired by the success of the Dynamic Time Warping technique in speech recognition, 1NN Dynamic Time Warping (denoted as 1NN-DTW) is introduced into time series analysis [11].

Different from 1NN-EUC and 1NN-DTW, which take the whole series similarity into account, feature-based TS classifiers are constructed on the basis of either local shapelets or bag-of-patterns (BOP) of time series. Shapelets are usually defined as a set of subsequences of the original time series, which is regarded as the most discriminative features for classifying time series. According to the recent evaluation of existing TSC solutions [10], Shapelet Transform (ST) [12,13] is regarded as the most accurate shapelet-based method. In Reference [14], a decision tree is built based on the distance to a set of shapelets. And the Learning Shapelets (LS) approach is proposed in Reference [15], which generates optimal shapelets synthetically. However, the computation cost of the shapelet-based methods has always been the main concern that limits their application in TSC.

For the BOP branch of feature-based TSC solutions, Symbolic Aggregate approXimation (SAX) is regarded as the first published BOP approach. It transforms the raw time series into a sequence of characters by using a fixed-length sliding window and then employs the 1NN classifier based on a self-defined distance between two character sequences for classification [16]. SAX is extended to SAX-VSM [17] by combining tf-idf weighted features with Cosine distance to obtain a single feature vector for each class, which not only saves memory space but also speeds up the execution time. The TS Bag-of-features Framework (TSBF) [18] is another member of the BOP family. The idea of TSBF is to build a supervised codebook generated by a random forest classifier with random-length windows at random positions. The BOP-based model BOSS (Bag-of-SFA-Symbols) [19] is currently the most accurate BOP-based approach by replacing SAX with Symbolic Fourier Approximation (SFA) [22]. And WEASEL [23] is proposed to achive fast and accurate classification on time series by extracting smaller but yet discriminative features from the original time series.

To further enhance accuracy, ensemble solutions are designed by incorporating different types of core classifiers and making the final decisions based on techniques, such as majority voting, bagging, or weighted aggregation. Elastic Ensemble (EE) [24] employs 11 core classifiers, while COTE [25] combines 35 core classifiers, including EE. The performance of the ensemble TSC solutions depends much on the variety of core classifiers and the decision-making strategy.

Recently, there have been attempts to apply deep learning models for solving TSC problems. In Reference [20], for instance, several types of deep learning models are directly applied to time series classification without any preprocessing. According to the experimental evaluation in Reference [20], FCN performs the best among all tested deep learning models, which shows competitive performance to COTE and BOSS.

## 6. Conclusions

To perform classification on diverse types of time series data in a timely manner without costly hand-crafting feature engineering, in this paper, we propose a new framework, *Edge4TSC*, that allows time series to be processed by deep learning classifiers on the edge device. A new time series representation is designed by utilizing the binary distribution tree and integrated with the proposed framework. By conducting experiments for 4 classifiers using the raw or proposed BDT-based representation over 6 challenging datasets, we validated the potential of the proposed representation for further enhancing classification accuracy. And by comparing the time consumption of classifier training on the end device and the edge device, the offloading mode is verified to be effective for dramatically speeding up the classifier training process. Furthermore, a comprehensive analysis of the impact of key factors (i.e., sr, bins, and level) was performed to offer deep insights into key parameter tuning strategies. In addition, as mentioned in Section 3, the BDT-based representation could be seamlessly integrated with any classifier that is able to handle vectorized representations.

## Figures and Tables

**Table 1:** Parameters of Binary Distribution Tree (BDT)-based representation.

Symbol	Description	Range
sr	Split ratio for determining the split position for the current node.	(0.00, 1.00)
bins	Number of bins for calculating the width of each bin.	(1, *∞*)
level	Level of BDT that all nodes from level 0 to this level are concatenated to generate the representation, and L is the total number of BDT levels.	[0, L)

**Table 2:** Datasets description.

Dataset	No. of Classes	Train/Test Size	Series Length	Domain
Haptics	5	155/308	1092	Passgraph Identification
Herring	2	64/64	512	Otolith Analysis
InlineSkate	7	100/550	1882	In-Line Speed Skating
InsectWingbeatSound	11	220/1980	256	Flying Insect Classification
Phoneme	39	214/1896	1024	Phoneme Classification
ScreenType	3	375/375	720	Screen Type Identification

**Table 3:** Overall TSC results.

Dataset	1NN		MLP		ResNet		FCN	
	RAW	BDT	RAW	BDT	RAW	BDT	RAW	BDT
Haptics	0.370	**0.435**	0.419	**0.468**	0.377	**0.445**	0.334	**0.432**
Herring	0.516	**0.750**	0.594	**0.734**	0.625	**0.734**	0.422	**0.719**
InlineSkate	0.342	**0.413**	0.336	**0.404**	0.187	**0.413**	0.187	**0.405**
InsectWingbeatSound	0.562	**0.573**	0.618	**0.623**	0.505	**0.568**	0.244	**0.516**
Phoneme	0.109	**0.161**	0.087	**0.190**	**0.319**	0.249	**0.249**	0.226
ScreenType	0.360	**0.461**	0.397	**0.477**	**0.605**	0.475	**0.619**	0.464
Average Accuracy	0.377	**0.466**	0.409	**0.483**	0.436	**0.481**	0.342	**0.46**
Standard Deviation	0.159	0.194	0.193	0.187	0.172	0.162	0.158	0.160
Win	0	6	0	5	2	4	2	4
Tie	0	0	1	1	0	0	0	0
Lose	6	0	5	0	4	2	4	2

Win: if the accuracy is at least 0.01 higher; Loss: if the accuracy is at least 0.01 lower; Tie: otherwise.

## Abbreviations

The following abbreviations are used in this manuscript:

TSC	Time Series Classification
BST	Binary Segment Tree
BDT	Binary Distribution Tree
1NN	1 Nearest Neighbor
MLP	Multi-Layer Perceptron
FCN	Fully Convolutional Network
ResNet	Residual Network
